# Case report: Safety of Tumor Treating Fields therapy with an implantable cardiac pacemaker in a patient with glioblastoma

**DOI:** 10.3389/fonc.2024.1441146

**Published:** 2024-08-22

**Authors:** Gregory B. Biedermann, Kathleen Merrifield, Leonardo Lustgarten

**Affiliations:** ^1^ Department of Radiation Oncology, University of Missouri, Columbia, MO, United States; ^2^ Department of Global Medical Safety, Novocure Inc., New York, NY, United States; ^3^ Department of Medical Affairs, Novocure Inc., New York, NY, United States

**Keywords:** TTFields therapy, Optune-Gio, cardiac pacemaker, glioblastoma, safety, active implantable medical device

## Abstract

Tumor Treating Fields (TTFields) therapy is an anti-cancer treatment modality that is delivered noninvasively to the tumor site via skin-placed arrays. The therapy is US Food and Drug Administration (FDA) approved and Conformité Européenne (CE) marked for adults with newly diagnosed and recurrent glioblastoma (GBM) (grade 4 glioma in the European Union). To date, there are limited data on the safety and efficacy of TTFields therapy in patients with implanted cardiac pacemakers. Herein, we report a case of a 79-year-old male patient with GBM receiving TTFields therapy with a prior medical history of cardiac events necessitating a cardiac pacemaker. The patient presented to the emergency department in May 2021 with newly onset left-sided weakness along with seizures. Based on an initial evaluation and results of the initial computed tomography (CT) scans (May 2021), the patient was clinically diagnosed with a high-grade glioma which was later confirmed as *IDH* wildtype following a biopsy. He was treated with radiotherapy (40 Gy in 15 fractions), followed by adjuvant temozolomide (TMZ) (75 mg/m^2^). TTFields therapy was initiated alongside maintenance TMZ (150 mg/m^2^). Average TTFields therapy usage was 67% throughout the duration of treatment. Follow-up CT scans (February and May of 2022) indicated stable disease. CT scans in August 2022 showed an increase in size of a mass with heterogeneous contrast enhancement and the patient subsequently passed away in October 2022. The patient’s last cardiac tests demonstrated that the pacemaker was operational with adequate cardiac function. This report suggests that TTFields therapy concomitant with an implanted electronic device may be safe in patients with GBM.

## 1 Introduction

Glioma is the most common primary malignant brain tumor with approximately 100,000 people diagnosed annually worldwide (1). Glioblastoma (GBM) remains the most challenging brain tumor with the lowest survival rates due to insufficient response to conventional combined therapy (2–4).

**Figure 1:**
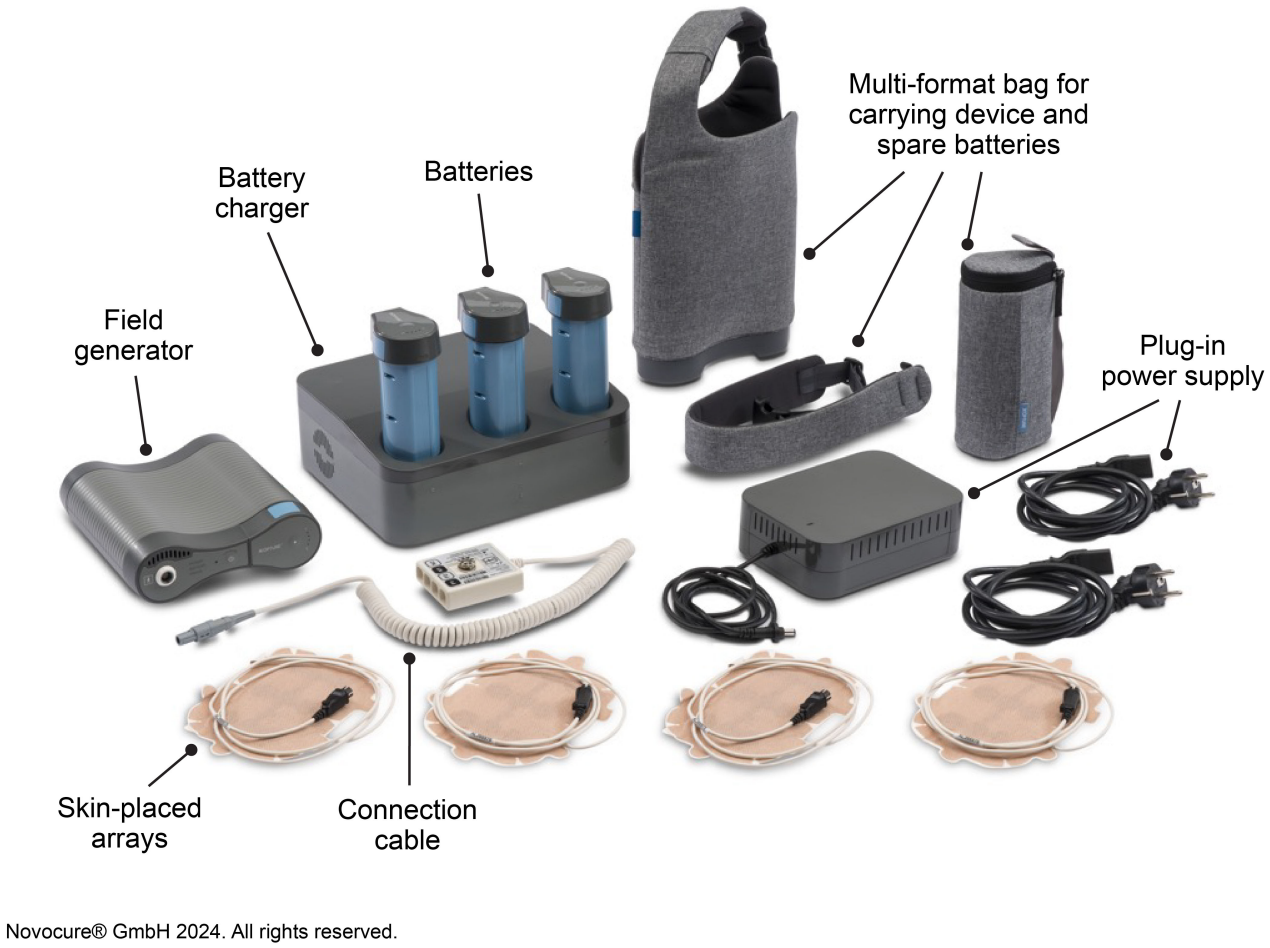
The NovoTTF-200A System.

Tumor Treating Fields (TTFields) therapy is a noninvasive, loco-regionally applied, anti-cancer treatment modality US Food and Drug Administration (FDA) approved for adults with newly diagnosed and recurrent GBM and Conformité Européenne (CE) marked for grade 4 glioma in Europe (5–8). Alternating TTFields therapy with temozolomide (TMZ; concurrent and adjuvant) and standard radiotherapy is a Category 1, preferred treatment recommendation for newly diagnosed GBM in the National Comprehensive Cancer Network Clinical Practice Guidelines in Oncology (NCCN Guidelines^®^) for Central Nervous System Cancers Version 1.2023 (9). TTFields are low intensity intermediate frequency, alternating electric fields generated by a portable medical device (**Figure 1**), and are delivered noninvasively to the tumor site via arrays placed on the skin. The electric fields act selectively on cancer cells due to their unique properties and exert physical forces to disrupt cellular processes crucial for cell viability and progression (10–12).

The first approval for TTFields therapy was granted by the FDA in 2011 for the treatment of recurrent GBM, following the results of the randomized, pivotal (phase 3) EF-11 clinical study (NCT00379470). EF-11 demonstrated comparable efficacy outcomes and improved quality of life with TTFields therapy compared with physician’s best choice of chemotherapy (8). TTFields therapy had a tolerable safety profile with low device-associated systemic toxicity, and most common adverse events (AEs) were localized, mostly resolvable, mild-to-moderate skin reactions (8). In 2015, additional approval was granted based on results of the randomized, pivotal (phase 3) EF-14 clinical study (NCT00916409) for TTFields therapy in newly diagnosed GBM with maintenance TMZ after standard chemoradiation (7, 13). EF-14 demonstrated statistically significant improvement in overall survival and long-term (5-year) survival versus TMZ alone (7, 13). Similar to EF-11, there was no added systemic toxicity associated with TTFields therapy, and the most common device-associated AEs were localized, mild-to-moderate skin reactions (7, 13). TTFields therapy usage has been shown to positively correlate with increased survival outcomes (14, 15). Therefore, in order to obtain maximum efficacy benefit, it is recommended that the TTFields device is worn for at least 18 hours per day (≥75% daily usage) (6, 14, 15).

Patients with GBM and cardiac comorbidities requiring a cardiac pacemaker often represent challenges for the treating physician. When treating such patients, it is important to ensure the continued functionality of the pacemaker, as well as address other comorbidities that can further complicate a patient’s treatment (5). Current regulatory labeling provides guidance to avoid TTFields therapy use in patients who have active implantable medical devices (AIMD) such as pacemakers, defibrillators, deep brain stimulators, spinal cord stimulators, or programmable ventriculoperitoneal shunts due to insufficient data regarding potential interference with the function of an AIMD (6, 16). As there is limited data on the concomitant use of TTFields therapy with AIMDs in patients with GBM, further research is needed to assess safety and tolerability in this patient population.

Here, we present the case of a 79-year-old male patient with GBM and a cardiac pacemaker receiving TTFields therapy. The uniqueness of this case is the provision of important safety information on the daily clinical application of TTFields therapy to a patient with GBM and an implanted cardiac pacemaker. Written informed consent was obtained from the patient’s next of kin for the publication of their medical case and accompanying images.

## 2 Case report

We report the case of a 79-year-old male patient with GBM with a prior medical history of cardiac and metabolic events (coronary artery bypass graft surgeries, multiple stents, atrial fibrillation, hypertension, hyperlipidemia, and type 2 diabetes). A full timeline of the episode of care is shown in **Supplementary Figure 1**. The patient had a dual chamber Boston Scientific cardiac pacemaker placed in 2014 at the age of 72 years, for symptomatic sinus node dysfunction with chronotropic intolerance. He also harbored a significant history of cancer, with an atypical melanocytic proliferation in the left iris consistent with uveal melanoma, which was excised in February 2020, with no known recurrence.

**Figure 2:**
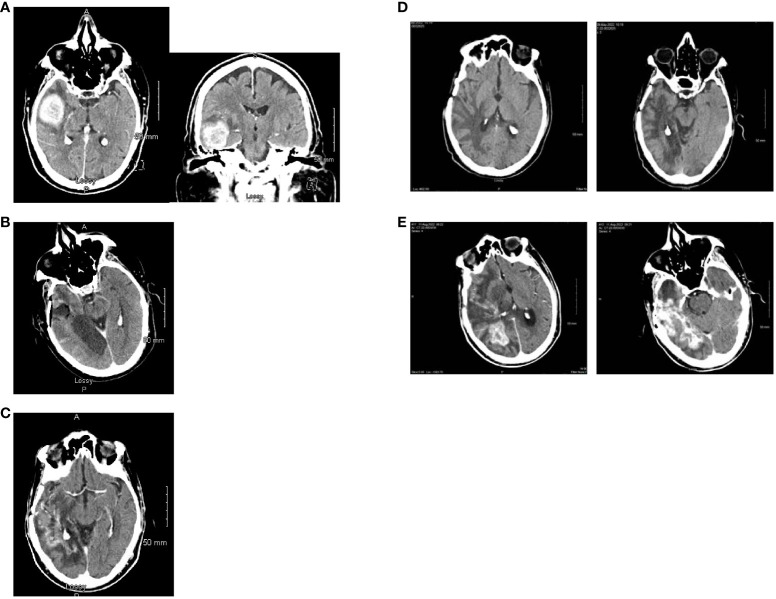
Computed tomography scan images. **(A)** Axial and coronal views at the time of initial diagnosis (May 2021) showing an avidly enhancing intra-axial mass in the right temporal lobe (maximal dimensions of 4.7 x 3.4 x 3.5 cm) with moderate surrounding vasogenic edema. **(B)** Post-operative axial computed tomography (May 2021). **(C)** Axial view with contrast enhancement while on treatment with Tumor Treating Fields (February 2022) showing stable disease. **(D)** Two slices of CT Axial views while on treatment with Tumor Treating Fields (May 2022) showing stable disease. No evidence of tumor is noted. **(E)** Two different level slices of CT Axial views. There is heterogenous and irregular contrast enhancement extending to the posterior right temporal lobe with vasogenic edema consistent with recurrence (August 2022).

**Figure 3:**
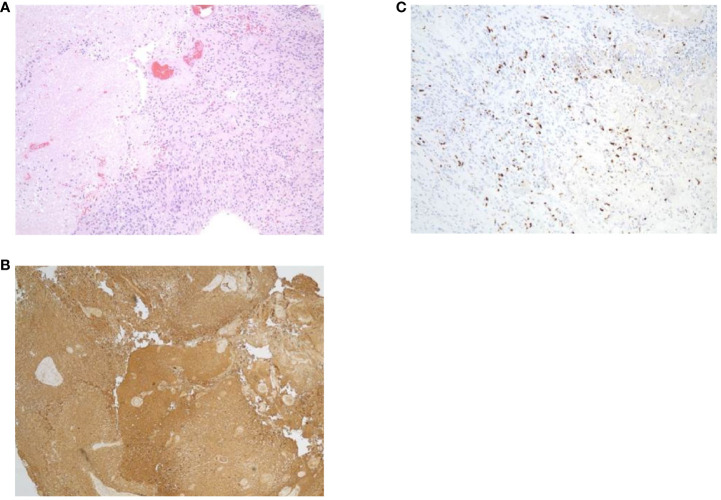
Specimens from pathology report. **(A)** Hematoxylin and eosin staining. **(B)** Glial fibrillary acidic protein. **(C)** Ki67.

In May of 2021, the patient presented to the emergency department with new onset left-sided weakness along with seizures. Upon admission, his initial computed tomography (CT) scans revealed a right-sided temporal lobe mass (4.7 x 3.4 x 3.5 cm) suggestive of malignancy (**Figure 2A**) and he underwent a right temporal craniotomy for subtotal tumor resection (**Figure 2B**). The frozen specimen was consistent with GBM, and the final pathology confirmed a World Health Organization grade 4 GBM (2021 classification; **Figures 3A–C**). Molecular profiling revealed the tumor to be *IDH* wildtype (**Table 1**).

**Table 1:** Tumor molecular pathology at diagnosis.

Parameter	Result
*IDH1 R132H* immunohistochemistry	Negative for mutation
*IDH1/2* sequencing	Negative for targeted mutations
*ATRX*	Retained expression
*p53*	Increased expression in infrequent tumor nuclei
*Ki67* proliferation index	15% (focal)
*MGMT* promoter	Indeterminate methylation result

Following diagnosis, the patient was treated with hypofractionated intensity-modulated radiotherapy (40 Gy in 15 fractions), which was completed in June 2021, along with concurrent daily TMZ at a dose of 75 mg/m^2^. TTFields therapy was initiated in August 2021 with maintenance TMZ (150 mg/m^2^, administered on day 1–5 of each 28-day cycle). TMZ was continued until January 2022. The patient continued to receive TTFields therapy until August 18, 2022, with decreasing overall daily usage over time.

Follow-up CT scans in February (**Figure 2C**) and May (**Figure 2D**) of 2022 were indicative of stable disease. The following CT scan in August 2022 (**Figure 2E**) revealed a significant increase in size of heterogeneously enhancing mass within the right temporal occipital lobe with surrounding vasogenic edema (11 mm left midline shift and mild right subfalcine herniation). Subsequently, the patient entered hospice care, and passed away in October 2022. The last pacemaker diagnostic report in October 2022 demonstrated overall adequate function of the pacemaker, with no evidence of malfunction. The patient’s last echocardiogram was conducted in May 2021 and showed a left ventricular ejection fraction of 50–55%, normal wall motion, and aortic sclerosis.

Total duration of TTFields therapy was 373 days with an average usage of 67% (range: 50–86%) over the entirety of treatment. In the months following therapy initiation (August–December 2021), daily usage ranged from 70–86%. In January of 2022, daily usage decreased to 60–65% due to skin irritation. During the final months of TTFields therapy (March–August 2022), average usage was approximately 50% and was well tolerated. TTFields therapy was eventually discontinued in August of 2022, per patient request to switch from active therapy to palliative care upon a decline in overall neurological function due to disease progression.

## 3 Discussion

AIMD, including pacemakers, are known to be susceptible to interference from electromagnetic fields. As both TTFields therapy and cardiac pacemakers work under the electromagnetic spectrum, it is not surprising there is concern among clinicians regarding their simultaneous clinical use.

TTFields therapy utilizes electric fields in a frequency range of 100–500 kHz, a range which is too high to stimulate muscle/nerve tissue and too low to have ionizing or significant heating effects (12, 17). The fields act via a multimodal mechanism of action, mainly by the application of electrical forces on charged polar components within cancer cells, thereby disrupting biological processes that are essential for cell viability and function (12, 18–22). The frequency of the fields is unique to the type of cancer cell being targeted, and they act selectively on cancer cells, without affecting the function of healthy cells (23). TTFields are electric fields and, although the therapy generates a low-level magnetic field, it is negligible (24) and not expected to have a relevant impact on AIMDs. Patients with AIMDs have been excluded from previous studies evaluating the safety and efficacy of TTFields therapy.

Cardiac pacemakers are capacitors where electric charge is stored at a potential of about 90 mV (25). They release electrical pulses that last 0.5–25 ms with a voltage of 0.1–15 volts at a frequency of up to 300 pulses per minute (5 Hz). The pacemaker takes measurements of the voltage (millivolts) generated by the heart when the heart contracts and sends electrical currents (milliamp) to set the pace of the heart. Pacemakers are typically categorized as external (temporary) or internal (implantable), with internal pacemakers usually being permanent and significantly more complex. It is estimated that 500,000–3,000,000 people in the United States have an implanted cardiac pacemaker, the majority of whom are >65 years old (25). This is precisely the typical age group where GBMs are most frequent (26). With an aging population and increasing life expectancy, the number of patients with GBM with cardiac pacemakers is expected to increase in the future, making it more prudent to explore the feasibility of TTFields and cardiac pacemakers.

Despite recent advances in systemic and targeted treatment options for GBM, there remains a significant unmet need for novel therapeutic options to improve survival in this difficult-to-treat patient population (27). TTFields therapy has demonstrated efficacy and safety data in patients with recurrent GBM (8) and newly diagnosed GBMs (7, 13, 15). The EF-14 study (newly diagnosed GBM; NCT00916409) demonstrated significant improvements in progression-free survival (6.7 months vs 4.0 months) and overall survival (20.9 months vs 16.0 months) for TTFields therapy concomitant with TMZ compared with TMZ alone, respectively (7). Five-year overall survival rates were significantly higher with TTFields therapy concomitant with TMZ compared with TMZ alone (5% vs 13%; *p* = 0.04) (7). TTFields also demonstrated comparable efficacy and favored toxicity and quality of life in the EF-11 study, which compared TTFields therapy with the best standard of care in patients with recurrent GBM (7). Clinical and real-world data demonstrate that TTFields therapy has a favorable safety profile, most commonly characterized as dermatologic AEs, with a low risk of device-related systemic AEs compared with chemotherapeutic regimens (7, 8, 28, 29).

As there is limited information on the potential interference between TTFields and AIMDs, current regulatory guidance advises against the use of TTFields therapy in patients who have AIMDs (6, 16). In this context, the current case study provides a valuable preliminary insight into the feasibility of TTFields therapy in a patient with GBM and a cardiac pacemaker. The patient in this case study had a dual chamber Boston Scientific cardiac pacemaker *in situ* for approximately 7 years prior to initiating TTFields therapy in August 2021. Importantly, no adverse influence on the overall function of the pacemaker was detected during the 12 months of TTFields therapy use.

The current findings on the lack of impact of TTFields therapy on pacemaker function are supported by recent reports in patients with GBM which also did not detect any interference between TTFields and AIMDs (28–32). In a post-market surveillance safety data study, there were no malfunctions reported among 49 patients who had non-programmable shunts (n = 44), pacemakers/defibrillators (n = 3) or programmable shunts (n = 2) (30). Similarly, a review of the clinical information for patients with GBM treated with TTFields in the US between November 2011 and June 2017 identified 50 patients with non-programmable shunts, five with programmable shunts, and five with pacemakers/defibrillators (31). The safety data obtained during post-marketing surveillance for all 60 patients were analyzed and did not reveal any new safety concerns on concurrent use of TTFields therapy with implanted devices (31). Furthermore, no arrhythmia or other cardiac AEs in the five patients with pacemakers/defibrillators was detected (31). An additional post-marketing safety study from November 2012–April 2021 with data including 156 patients with GBM who had implanted ventriculoperitoneal shunts (programmable and non-programmable) found that TTFields therapy was safe and did not interfere with effectiveness of the patient’s ventriculoperitoneal shunts (33). Real world data from a reported registry (PriDe registry) and several large retrospective studies of >25,000 patients with GBM (recurrent and newly diagnosed GBM) spanning more than a decade showed a consistent safety profile across various subgroups (including geriatric populations) (28, 29, 32). As expected, this real-world evidence included “off label” use of TTFields under different scenarios (e.g., in pediatric populations) without evidence of any new safety warning signals, which would have been captured by post-market safety surveillance.

The interpretation of case studies is limited as they represent an individual patient’s experience only, and the findings may not always be generalizable to broader patient populations. However, this case report represents a fairly typical real-world case of an elderly male patient with GBM, *IDH* wildtype (which is representative of approximately 90% of GBMs) (34), and significant comorbidities requiring an AIMD. This patient experienced a good response to TTFields therapy (stable disease which progressed once TTFields therapy was stopped) despite lower than recommended usage of ≥75% (16), and showed no clinical nor electrocardiographic signs of cardiac pacemaker malfunction while both devices were active concurrently.

The findings reported here, along with evidence from previously conducted retrospective studies, support the safety and feasibility of TTFields therapy among patients with GBM and AIMDs such as cardiac pacemakers. Prospective studies designed to specifically address the safety and efficacy of TTFields therapy in patients with AIMDs are feasible, but they present several challenges and considerations. Conversely, it may be more practical to evaluate cohorts of patients with AIMDs who are already enrolled in clinical studies as an exploratory endpoint. Clinical use of TTFields therapy in patients with AIMDs can be considered on a case-by-case basis by the treating clinician according to risk profiles of TTFields therapy use in patients with GBM.

## 4 Conclusion

Based on the case study reported here, concomitant use of TTFields therapy with an AIMD did not lead to any interference or malfunction, and the patient was able to derive benefit from both devices. Taken together with post-market safety and other real-world data, this suggests that the application of TTFields therapy may be safe among patients with GBM with implanted electronic devices such as cardiac pacemakers. These results warrant further evaluation of the use of TTFields therapy among patients with GBM and implanted electronic devices, which constitute an important subgroup of patients that can significantly benefit from TTFields therapy.

## Data availability statement

The original contributions presented in the study are included in the article/**Supplementary Material**. Further inquiries can be directed to the corresponding author.

## Ethics statement

Written informed consent was not obtained from the individual(s) for the publication of any potentially identifiable images or data included in this article because the individual passed away. Written informed consent was obtained from the individual’s next of kin for the publication of any potentially identifiable images or data included in this article.

## Author contributions

GB: Conceptualization, Data curation, Formal analysis, Investigation, Methodology, Project administration, Resources, Software, Supervision, Validation, Visualization, Writing – original draft, Writing – review & editing. KM: Conceptualization, Data curation, Formal analysis, Investigation, Methodology, Project administration, Resources, Software, Supervision, Validation, Visualization, Writing – original draft, Writing – review & editing. LL: Conceptualization, Data curation, Formal analysis, Investigation, Methodology, Project administration, Resources, Software, Supervision, Validation, Visualization, Writing – original draft, Writing – review & editing.
