# Supplementary material for: Case report: Safety of Tumor Treating Fields therapy with an implantable cardiac pacemaker in a patient with glioblastoma
Source: Front Oncol. 2024 Aug 22;14:1441146. doi: 10.3389/fonc.2024.1441146 (PMC11374662; doi:10.3389/fonc.2024.1441146)
Supplement: Supplementary file 1 [file DataSheet1.docx]

SUPPLEMENTARY FIGURE 1. Timeline of Case Study


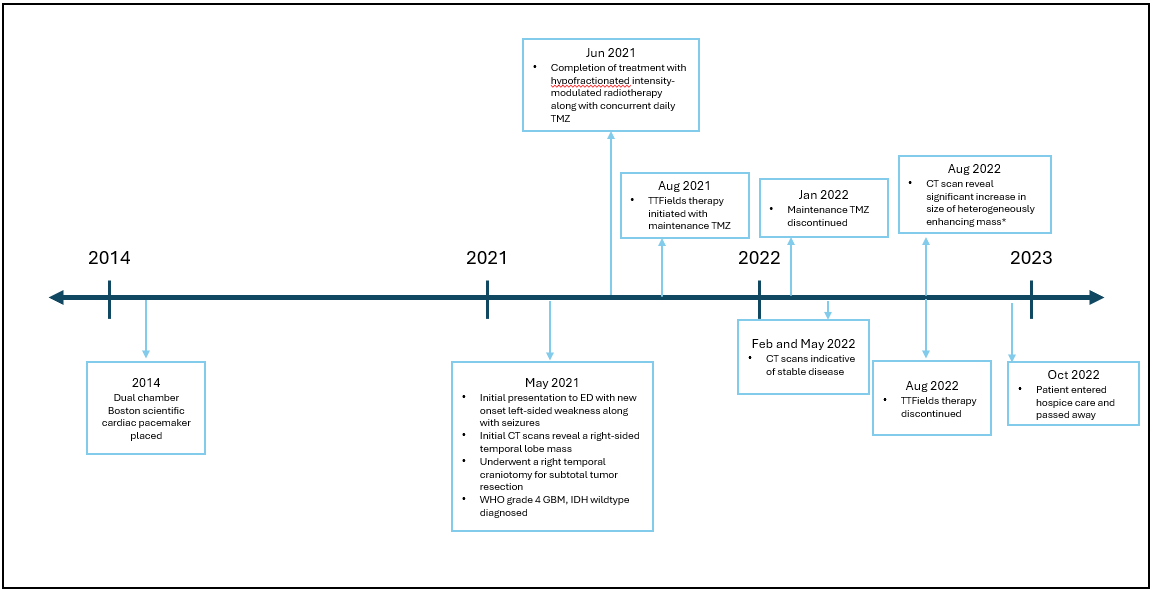


^*^Within the right temporal occipital lobe with surrounding vasogenic edema.

CT, computer tomography; ED, emergency department; IDH, Isocitrate dehydrogenase; TTFields, Tumor Treating Fields; TMZ, temozolomide; WHO, World Health Organization.
